# Generation and Efficacy Evaluation of Recombinant Classical Swine Fever Virus E2 Glycoprotein Expressed in Stable Transgenic Mammalian Cell Line

**DOI:** 10.1371/journal.pone.0106891

**Published:** 2014-09-08

**Authors:** Rong-Hong Hua, Hong Huo, Ye-Nan Li, Yao Xue, Xiao-Lei Wang, Li-Ping Guo, Bin Zhou, Yong Song, Zhi-Gao Bu

**Affiliations:** 1 State Key Laboratory of Veterinary Biotechnology, Harbin Veterinary Research Institute, Chinese Academy of Agricultural Sciences, Harbin, Heilongjiang, People's Republic of China; 2 College of Life Science, Heilongjiang University, Harbin, Heilongjiang, People's Republic of China; 3 Key Laboratory of Animal Diseases Diagnosis and Immunology, Ministry of Agriculture, College of Veterinary Medicine, Nanjing Agricultural University, Nanjing, Jiangsu, People's Republic of China; University of Massachusetts Medical Center, United States of America

## Abstract

Classical swine fever virus (CSFV) is the causative agent of classical swine fever (CSF), which is a highly contagious swine disease that causes significant economic loses to the pig industry worldwide. The envelope E2 glycoprotein of CSFV is the most important viral antigen in inducing protective immune response against CSF. In this study, we generated a mammalian cell clone (BCSFV-E2) that could stably produce a secreted form of CSFV E2 protein (mE2). The mE2 protein was shown to be N-linked glycosylated and formed a homodimer. The vaccine efficacy of mE2 was evaluated by immunizing pigs. Twenty-five 6-week-old Landrace piglets were randomly divided into five groups. Four groups were intramuscularly immunized with mE2 emulsified in different adjuvants twice at four-week intervals. One group was used as the control group. All mE2-vaccinated pigs developed CSFV-neutralizing antibodies two weeks after the first vaccination with neutralizing antibody titers ranging from 1∶40 to 1∶320. Two weeks after the booster vaccination, the neutralizing antibody titers increased greatly and ranged from 1∶10,240 to 1∶81,920. At 28 weeks after the booster vaccine was administered, the neutralizing antibody titers ranged from 1∶80 to 1∶10240. At 32 weeks after the first vaccination, pigs in all the groups were challenged with a virulent CSFV strain at a dose of 1×10^5^ TCID_50_. At two weeks after the challenge, all the mE2-immunized pigs survived and exhibited no obvious symptoms of CSF. The neutralizing antibody titer at this time was 20,480. Unvaccinated pigs in the control group exhibited symptoms of CSF 3–4 days after challenge and were euthanized from 7–9 days after challenge when the pigs became moribund. These results indicate that the mE2 is a good candidate for the development of a safe and effective CSFV subunit vaccine.

## Introduction

Classical swine fever (CSF), which is caused by the CSF virus (CSFV), is a highly contagious severe and often fatal disease of pigs. CSFV is a member of the *Pestivirus* genus and the Flavivirade family [1]. The CSFV genome consists of a single-stranded, positive-sense RNA with a single open reading frame (ORF) encoding a polyprotein which is cleaved into 11 mature viral proteins. Of these 11 proteins, four proteins including nucleocapsid protein C and three envelope glycoproteins E^rns^, E1, and E2 are structural proteins. E2 is the most immunodominant protein in the envelope and plays an important role in virus neutralization [2], [3].

E2 is the target of CSF subunit vaccine research, and has been expressed in baculovirus [4], [5], yeast [6], and adenovirus [7], [8] expression systems. In particular, many studies have investigated a baculovirus expression system expressing the E2 protein for use as a subunit vaccine. This expression system was found to be effective and has been licensed for commercial use [9]–[19]. As CSFV is a mammalian virus, the CSFV E2 expressed in mammalian cells would be more similar to its native conformation and glycosylation form. It is well known that inappropriate glycosylation can impact the immunogenicity. Therefore, the E2 protein expressed in a mammalian system would provide better levels of immunogenicity for the induction of protective immunity. Currently, no researchers have developed a CSF subunit vaccine by expressing the E2 protein in a mammalian cell line.

Therefore, in this study, we aimed to develop a CSF subunit vaccine by expressing the E2 protein in a mammalian cell line. To this end, we generated a new cell line, BCSFV-E2, using BHK-21 cells as the parent cell line, and these cells could stably produce and secrete a homodimer of glycosylated E2 protein (mE2). We then immunized pigs with this antigen and tested their immunity against CSFV infection.

## Materials and Methods

### Ethics statement

Care of laboratory animals and animal experimentation were performed in accordance with animal ethics guidelines and approved protocols. All animal experiments were approved by the Animal Ethics Committee of Harbin Veterinary Research Institute of the Chinese Academy of Agricultural Sciences.

### Cells and viruses

Baby hamster kidney cells (BHK-21; American Type Culture Collection CCL-10) and porcine kidney cells (PK-15; American Type Culture Collection CCL-33) were cultured at 37°C in a 5% CO_2_ atmosphere in Dulbecco's modified Eagle's medium (DMEM; Gibco, Invitrogen, Carlsbad, CA) supplemented with 10% fetal bovine serum (FBS; Gibco, Grand Island, NY), 100 U/ml penicillin, and 100 µg/ml streptomycin (Gibco, Grand Island, NY). The virulent Shimen strain of CSFV, which is the standard virulent strain that has been used for vaccine potency tests in China since the 1950s, was used in this study.

### Prokaryotic expression of CSFV E2 and generation of monoclonal antibodies against CSFV E2

The gene encoding the *N*-terminal end (340 aa) of the E2 protein was amplified from the virulent CSFV Shimen strain using reverse-transcriptase polymerase chain reaction (RT-PCR). The sequences of the upstream and downstream primers used for the amplification are 5′-TGTCCATGGGCCGGCTAGCCTGCAAGGAAG-3′ and 5′-ATAGGATCCTTATGCGAAGTAATCTGAGTGGCGGT 3′, respectively. The truncated E2 protein was expressed with an MBP tag and purified according to the procedure described previously [20]. The purified recombinant protein MBP-E2 was used as an immunogen in mice. Hybridomas secreting anti-E2 antibodies were generated according to the procedure described previously [20]–[22]. Ascitic fluid was generated in pristane-primed BALB/c mice. The monoclonal antibodies were purified by affinity chromatography in a HiTrap Protein G HP column (GE, Sweden) according to the manufacturer's instructions. The identities of the heavy and light chains of each mAb were determined using a Pierce Rapid Isotyping Kit with Kappa and Lambda Mouse (Thermo, Rockford, IL).

### Construction of plasmids carrying the CSFV E2 gene

First, a genetic codon-optimized CSFV cDNA encoding the viral signal peptide of the carboxyl terminus of the E1 and E2 proteins (amino acid positions 668–1029 with an additional methionine at the amino terminus **AAC68902.2**) of the Shimen strain was synthesized and cloned into the pUC57 plasmid. The pUC57-opti-CSFV-E2 plasmid was digested with *Sac*I and *Xho*I, and the target DNA fragment was inserted into the *Sac*I and *Xho*I sites of the expression vector pCAGneo [23] to generate pCAGneo-opti-CSFV-E2. The pCAGneo plasmid contains the neomycin resistance gene, which confers resistance to G418 (EMD Chemicals Inc., San Diego, CA, USA). The resulting plasmid pCAGneo-opti-CSFV-E2 was used to transfect stable cell lines.

### Establishment of stable cell lines constitutively producing the E2 antigen

On the day before transfection, BHK-21 cells were passaged and cultured in DMEM containing 10% FBS. Monolayer or subconfluent BHK-21 cells were transfected with the pCAGneo-opti-CSFV-E2 plasmid using FuGENE HD transfection reagent (Roche Diagnostic GmbH, Mannheim, Germany). One day after transfection, the cells were digested with trypsin-ethylenediamine tetraacetic acid (EDTA), transferred to new plates, and cultured in growth medium containing 1,000 µg/mL G418. After 24 h of culture, the adherent cells were digested and diluted to a concentration of approximately 1 cell/100 µL and transferred to 96-well plates. G418-resistant colonies were picked about 7–10 days later and transferred to 24-well plates. The concentration of the E2 antigen in the culture supernatants of wells with the resistant cells were determined by measuring the optical density (OD) at 450 nm and compared using a CSFV AG ELISA kit (Median Diagnostics Inc., Korea). The cell line (#12) with the highest OD_450_ value was selected and cloned by two more cycles of the limiting dilution method in G418-containing medium. One clone, which we designated E2–12, was selected and maintained in G418-supplemented medium for further characterization and antigen production.

### Indirect immunofluorescence assays and flow cytometry analysis

Cells were cultured to subconfluence in 24-well plates. The cells were then washed with phosphate-buffered saline (PBS), fixed with 4% paraformaldehyde at room temperature for 20 min, washed with PBS three times, and rinsed for 5 min after each wash. The cells were then permeated with PBS containing 0.1% Triton X-100 (PBS-T) at 4°C for 10 min, washed with PBS for 5 min at room temperature, blocked with PBS containing 4% BSA (PBS-B) at 37°C for 30 min, incubated with monoclonal antibodies against CSFV E2 (12C4; generated with recombinant CSFV E2 protein expressed in *Escherichia coli* as described above. Figure S1) at 37°C for 1 h, and washed with PBS-T three times. The cells were then incubated with fluorescein isothiocyanate (FITC)-conjugated goat anti-mouse antibodies (secondary antibody) at room temperature for 1 h, and then with 1 µg/mL of 4′,6-diamidino-2-phenylindole dihydrochloride (DAPI) solution for 15 min. The cells were then washed three times as described above and observed under a fluorescence microscope (IMT2 Olympus, Tokyo, Japan). For flow cytometry analysis, adherent cells were digested with trypsin-EDTA solution. The cell suspension was washed three times and centrifuged. Then cells were fixed and stained with 12C4 and FITC-conjugated goat anti-mouse antibodies. Flow cytometry was carried out on a BD FACSAria system (BD Biosciences).

### Immunoaffinity purification of recombinant mE2 protein

12C4 mAbs purified from ascites were coupled to 1.0 g of CNBr-activated Sepharose-4B (GE Healthcare Bio-Sciences AB, Sweden) according to the manufacturer's instructions. The beads were then packed in a PD-10 column (GE, Sweden). BCSFV-E2 cells were passaged and cultured in DMEM supplemented with 10% FBS. When a confluent monolayer was achieved, the medium was replaced with fresh medium to maintain the culture (DMEM with 1% FBS) for 4 to 5 days. As long as the live cells were confluent, the medium was harvested at 4- to 5-day intervals. The supernatant was then centrifuged at 4,000 rpm for 15 min and filtered through a 0.45-µm membrane, and 200 mL of the supernatant was allowed to flow through the Sepharose column by gravity. The column was then washed with PBS, and the bound mE2 was eluted with 0.1 M glycine-HCl (pH 2.7). The eluate was immediately neutralized with 1 M Tris-Cl (pH 9.0). The eluted fractions were resolved by sodium dodecyl sulfate-polyacrylamide gel electrophoresis (SDS-PAGE), and the protein concentration was determined with the BCA Protein Assay Kit (Thermo, IL).

### Antigen capture ELISA for quantification of the mE2 antigen

The mE2 titers were determined by a sandwich ELISA with the CSFV AG ELISA kit (Median Diagnostics Inc., Korea). The titers were presented as the highest dilution of the culture medium in which mE2 titers could be detected. For quantification of the mE2 antigen, the immunoaffinity-purified mE2 was serially diluted twofold starting from 300 to 4.6875 ng/mL and used as the standard in antigen capture ELISA. The mE2 concentrations were measured using a standard curve of serial dilutions of the Sepharose-purified mE2.

### *N*-linked glycosylation analysis and Western blot analysis

Culture supernatants were concentrated by centrifugation with centrifugal filter units (Millipore, Carrigtwohill Co., Ireland). The samples were mixed with one-fourth volume of 5×SDS sample loading buffer with or without β-mercaptoethanol and boiled for 10 min. For *N*-linked glycosylation analysis, the concentrated sample was digested with peptide: *N*-glycosidase F (PNGase, New England Biolabs) according to the manufacturer's instruction. The samples were analyzed by Western blot as described previously [22]. Briefly, samples were separated on Any kD Resolving Gel (Mino-PROTEAN TGX Precast Gels; Bio-Rad Laboratories, CA). The separated proteins were transferred onto a nitrocellulose membrane with a Transblot apparatus (Bio-Rad, CA). The membrane was washed three times in PBS-T with shaking, blocked with 5% skimmed milk at 4°C overnight, and incubated with 12C4 at 37°C for 1 h. The membranes were washed three times with PBS-T and incubated with anti-mouse Alexa Fluor 680-conjugated secondary antibodies (Invitrogen, Carlsbad, CA, USA) for 1 h at 37°C, after which they were washed three times with PBS-T. Protein bands were detected with the Li-Cor Odyssey system (Li-Cor Biosciences, NE).

### Immunization and challenge of pigs

The cell culture supernatants containing 30 µg/mL of mE2 antigen were mixed or emulsified with Montanide ISA 15A VG, Montanide ISA 206 VG, Montanide ISA 61 VG, and Montanide ISA 50V2 adjuvants (Seppic, France). For this experiment, 25 6-week-old Landrace pigs that were tested to be negative for CSFV antibodies were used. The pigs were randomly divided into five groups (groups 1–5) of five pigs each. Pigs in groups 1–4 received intramuscular injections of 2 mL of vaccine with different adjuvants. Group 1 received Montanide ISA 15A VG; group 2 received Montanide ISA 206 VG; group 3 received Montanide ISA 61 VG; and group 4 received Montanide ISA 50V2 adjuvants. Four weeks after the first immunization, the pigs received enhanced immunization using the same doses as the first immunization using the same protocol. The pigs in group 5 received intramuscular injections of 2 mL of PBS and served as the negative controls. Serum samples were obtained at 2, 4, 6, 8, 12, 16, 20, 26, 30, and 32 weeks after immunization. Thirty-two weeks after vaccination, three pigs were randomly selected from each group and challenged intramuscularly via the neck with 1×10^5^ TCID_50_ of the virulent CSFV strain in a volume of 2 mL. The pigs were observed daily for clinical signs, and rectal temperatures were measured. Blood samples were obtained from the pigs at days 6 and 9 post-challenge and used to inoculate PK-15 cells. Three days after inoculation, immunoperoxidase monolayer assay (IPMA) was used to detect the presence of CSFV in the cells [24]. Two weeks after the challenge, all the surviving animals were euthanized and subjected to pathological examination.

### Serological examination

Serum samples were tested for CSFV E2-specific antibodies by antibody blocking ELISA with a CSFV Antibody Test Kit (IDEXX, Switzerland) according to the manufacturer's instructions. The antibody titers were presented as the highest dilution of serum for which a blocking rate of ≥40% was obtained. The serum titers of virus-neutralizing antibodies were tested using a neutralizing peroxidase-linked antibody (NPLA) assay as described previously [24]. Briefly, serum samples were heat-inactivated at 56°C for 30 min. Then, sera were serially diluted two-fold and incubated with 200 TCID_50_ of the CSFV Shimen strain in DMEM with 5% FBS for 1 h at 37°C. Residual virus infectivity was determined by adding 1×10^4^ PK-15 cells to a 100-µL aliquot of the diluted serum samples and incubating the mixture at 37°C for 3 days. The cells were subjected to immunofluorescence staining with 12C4. Neutralizing antibody titers were expressed as the reciprocal of the highest dilution that caused 50% neutralization.

## Results

### Establishment of a stable cell clone continuously expressing CSFV-E2

**Figure 1:**
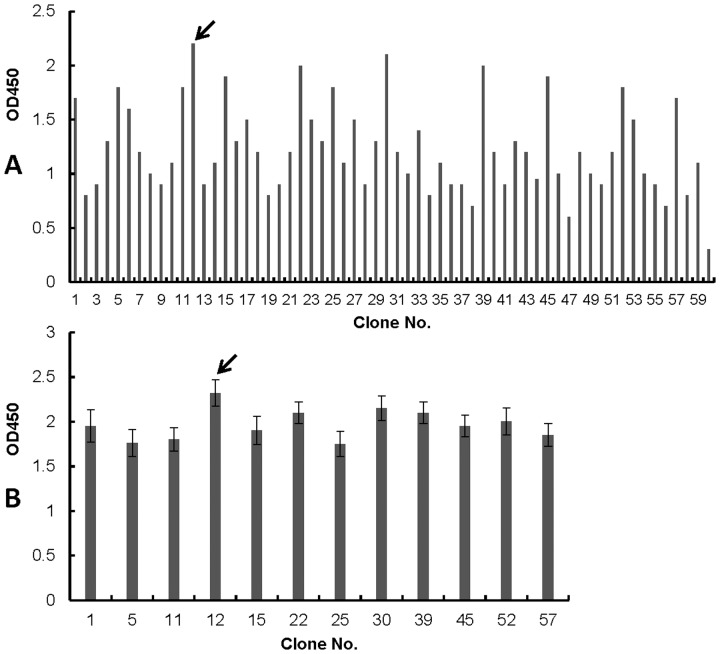
Selection and comparison of transfected cell clones expressing E2 protein using ELISA. E2 protein expressing titers were detected and compared between 60 G418-resistant and IFA-positive cell clones (A). Among the 60 cell clones, 12 cell clones with a relative high titer of E2 were selected and further compared using ELISA (B). The no. 12 cell clone, which had the highest ELISA titer value was selected for further characterization (as shown by the arrow).

**Figure 2:**
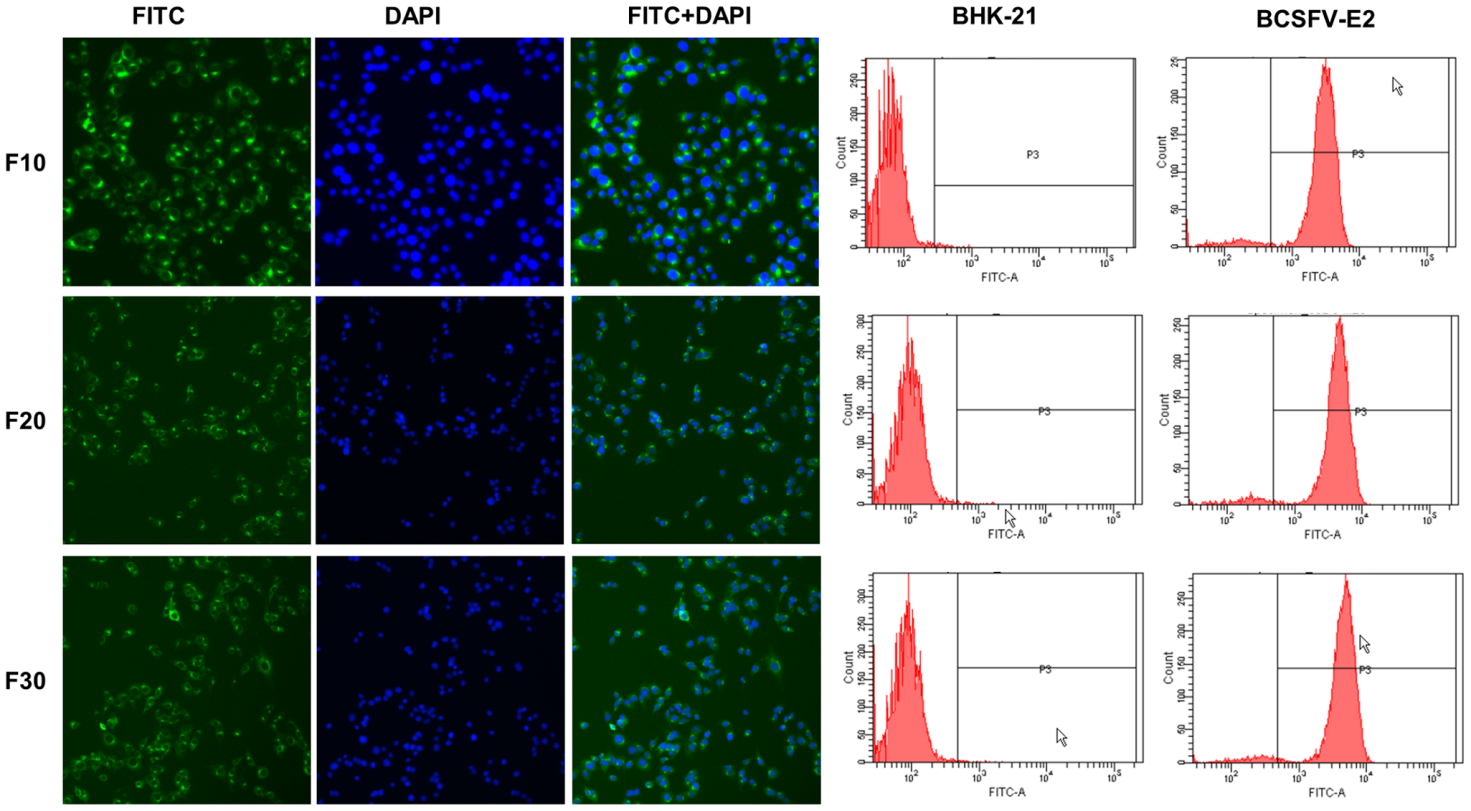
Immunofluorescence and flow cytometry analysis of BCSFV-E2 cell passages. The tenth (F10), twentieth (F20), and thirtieth (F30) generations of BCSFV cells were analyzed by indirect immunofluorescence and flow cytometry. Both assays show a high percentage of cells expressing CSFV E2.

G418-resistant and E2 protein-specific immunofluorescence staining-positive cell colonies were selected from the cloned colonies of pCAGneo-opti-CSFV-E2 plasmid-transfected BHK-21 cells and transferred to 24-well plates. Sixty clones were selected during the first round of selection by IFA. Among the 60 clones, twelve clones were selected for further comparison by ELISA. After the E2 protein levels in the culture medium of the cell clones were compared, we selected the cell clone with the highest E2 protein titer for further characterization (Fig. 1). After immunofluorescence analysis and further purification, the selected clone was designated as BCSFV-E2, and the antigen expressed by BCSFV-E2 cells was designated as mE2. The cells were cultured and maintained in G418-containing medium to observe mE2 antigen production. The morphology of the BCSFV-E2 cells was indistinguishable from that of the parental BHK-21 cells and induced no polykaryocyte formation. When the tenth passage of the BCSFV-E2 cell clone was examined by indirect immunofluorescence and flow cytometry analysis, nearly 100% and 94% of the cells were found to express mE2 by immunofluorescence and flow cytometry analyses, respectively (Fig. 2). This high efficiency of E2 expression was maintained for at least 30 passages.

**Figure 3:**
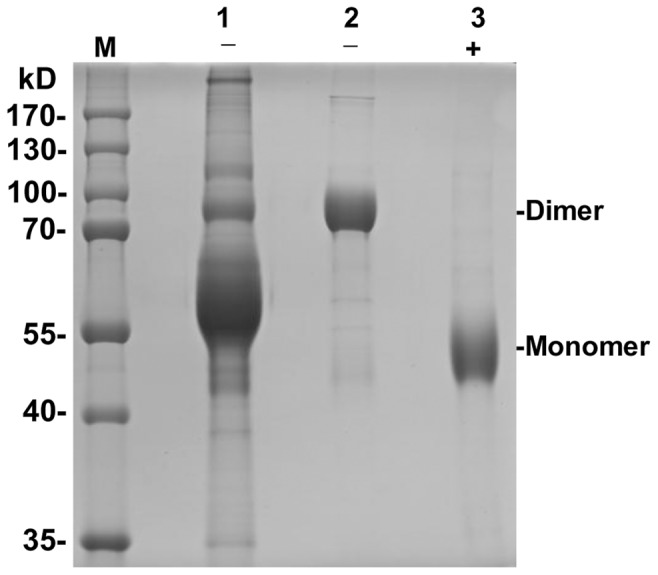
SDS-PAGE analysis of mE2 in culture and purified medium. M, Molecular marker; 1, supernatant of BCSFV-E2; 2 and 3, Immunoaffinity-purified mE2; –, non-reduced; +, reduced with β-mercaptoethanol.

**Figure 4:**
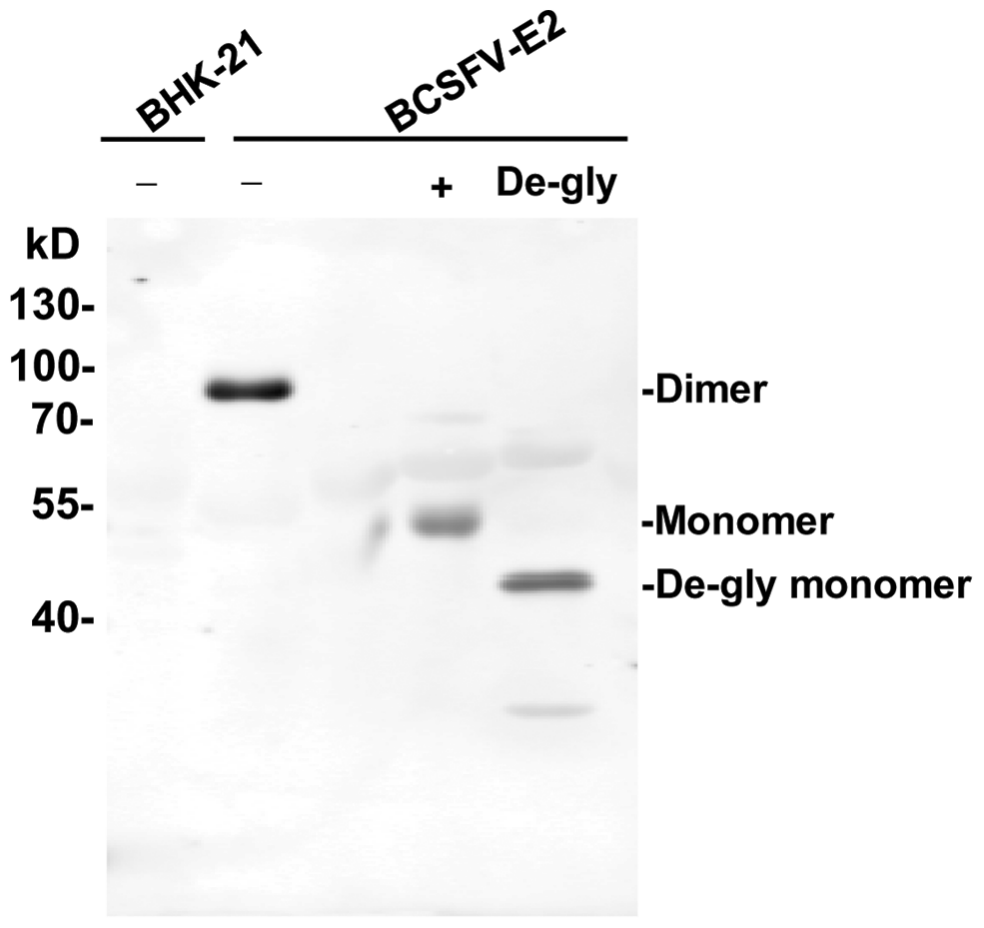
Western blot analysis of mE2 in culture supernatants. BHK-21 cells and BCSFV-E2 cells culture supernatants were separated in the absence (+) or presence (+) of β-mercaptoethanol or treated with peptide: *N*-glycosidase F (PNGase F) followed by Western blot analysis with 12C4 monoclonal antibody.

As the mE2 protein expressed in BCSFV-E2 cells did not contain the C-terminal transmembrane region, it could be secreted into the culture medium. The culture supernatant was harvested and purified with a Sepharose column chemically coupled with the 12C4 mAb. Results of the SDS-PAGE revealed that the molecular weight of purified mE2 under non-reducing condition had a molecular weight of approximately 90 kDa. In the presence of β-mercaptoethanol, the molecular weight of the 90-kDa band was reduced to approximately 45 kDa (Fig. 3). Thus, the 90- and 45-kDa bands represented the homodimer and monomer forms of the mE2 protein, respectively. The culture supernatant was analyzed by Western blot analysis. The 12C4 mAbs bound to the 90- and 45-kDa bands, obtained under the non-reducing and reducing conditions, respectively. In addition, the mE2 protein was sensitive to PNGase F enzyme. When treated with this enzyme, the molecular weight of the mE2 monomer changed from 45 kDa to approximately 42 kDa (Fig. 4).

### Expression of mE2 in BCSFV-E2 cells

**Figure 5:**
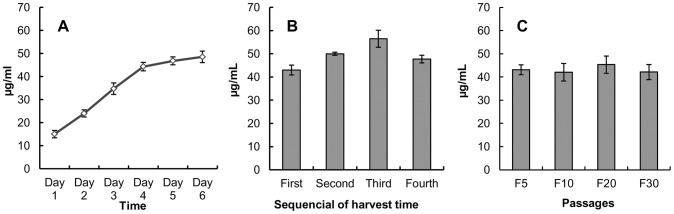
Production of mE2 by BCSFV-E2 cells. (A) Confluent BCSFV-E2 cells were incubated in maintenance medium for six days. ELISA was used to quantify the titers of mE2 antigen accumulated in the supernatant every day (24 h). (B) The culture medium of confluent BCSFV-E2 cells was harvested every 4–6 days and replaced with fresh medium, and the mE2 antigen titers in the harvested culture supernatants were determined by ELISA. (C) The culture supernatants of different passages of cell line were determined by ELISA.

The mE2 protein titers were examined within 15 passages in 6,500-cm^2^ spinner bottles after the cloned and purified BCSFV-E2 cells were cultured and cryopreserved. The BCSFV-E2 cells were transferred from flasks to spinner bottles. When the cells reached confluence, the culture medium (supplemented with 10% FBS) was replaced by maintenance medium (supplemented with 1% FBS) and incubated for 4–6 days. A 2-mL aliquot of the culture medium was collected every day, and the mE2 protein concentration in the medium was quantified by antigen capture ELISA. As shown in Figure 5A, the mE2 concentration in the culture medium kept increasing in the first four days. The concentration plateaued on day 4, after which it increased slightly from day 4 to day 6. The culture medium was harvested and replaced with fresh maintenance medium every five days for a total of four times, and mE2 protein concentrations in the harvested medium were determined by ELISA. Figure 5B shows a representative result of the mE2 concentrations in the medium harvested from the spinner bottles. The mE2 concentrations in all the harvested samples were found to be >40 µg/mL. Furthermore, the mE2 concentrations in the medium harvested the second and third times reached 50.0 and 56.5 µg/mL, respectively. During passages of the cell line in vitro, the amount of mE2 in the medium was also quantitatively detected using ELISA. The results showed that the cell line maintained a high level of expression efficiency for at least thirty passages (Fig. 5C).

### Immunogenicity and protective efficacy of the mE2 response in immunized pigs

**Figure 6:**
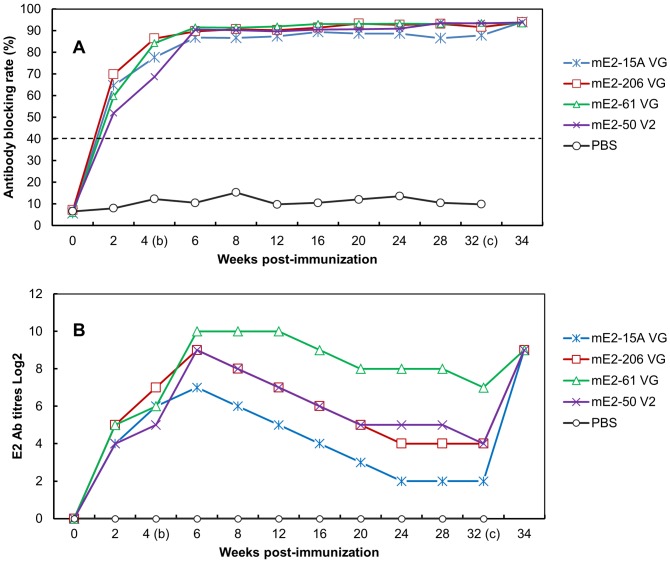
ELISA antibody development after vaccination and challenge infection. Pigs were vaccinated with mE2 antigen with four kinds of adjuvants. The pigs were given a booster vaccination four weeks after the first vaccination. The pigs were then challenged with a virulent CSFV strain at 32 weeks post-vaccination. Pigs inoculated with PBS were used as controls. The antibody titers are presented as blocking rates (A) and the reciprocal of the highest dilution of serum for which a blocking rate of ≥40% was obtained (B).

To evaluate the immunogenicity of the mE2 antigen, 6-week-old piglets were immunized with mE2 antigen emulsified with four types of adjuvants. All the mE2-immunized groups seroconverted to the CSFV-E2 two weeks after vaccination, and the blocking rates ranged from 52%–69.9%. At four weeks after vaccination, the blocking rates ranged from 68.7–86.4%. Two weeks after booster vaccination, the blocking rates of all the groups were >86%, and these high blocking rates lasted throughout the experiment (Fig. 6A).

The efficacy of immunity between the different adjuvant groups was compared by determining the CSFV E2 antibody titers by ELISA. As shown in Figure 6B, the E2 antibody titers of the four groups ranged from 16–32 at two weeks after vaccination. These values reached the highest level at two weeks after vaccination and ranged from 128–1024, and decreased thereafter at different rates in the different groups. At 32 weeks post-immunization, the antibody titers ranged from 4–128. At two weeks post-challenge, the antibody titers of all the four immunized groups reached 512.

**Figure 7:**
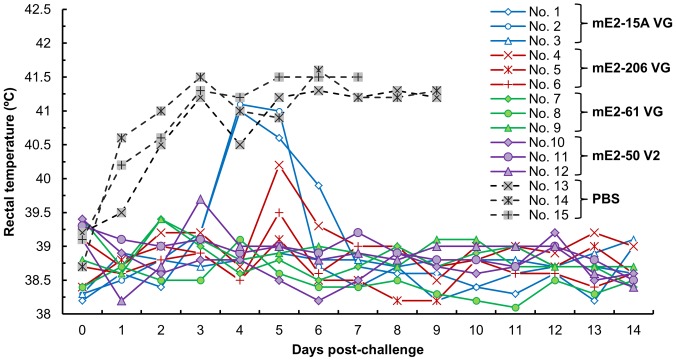
Rectal temperatures post-challenge. Vaccinated and control pigs were challenged with the virulent CSFV Shimen strain. After challenge, rectal temperatures of each pig were measured daily for 14 days.

All the immunized pigs developed neutralizing antibodies, and the NPLA titers ranged from 40–320 at two weeks post-immunization. A strong booster response was observed in all the immunized groups. Two weeks after the booster vaccination, the NPLA titers peaked and ranged from 10240–81920. At 32 weeks post-immunization, the NPLA titers ranged from 80 to 10420 (Table 1). At this time, three of five pigs in each group were challenged with a virulent strain of CSFV. All the vaccinated pigs were completely protected against lethal challenge. Except for two pigs in group mE2–15 VG that developed fever (≥40.5°C) that lasted for two days (Fig. 7), none of the pigs showed any signs of disease. Furthermore, no virus was detected in the blood of the challenged pigs (Table 2). In contrast, the three challenged pigs from the control group developed fever (≥40.5°C) at a high frequency of 23/25 (Table 2), and the control pigs exhibited severe signs of disease and were sacrificed at days 7 and 9 post-challenge when they became moribund.

**Table 1:** Neutralizing antibody response following vaccination and challenge infection.

	Neutralizing antibody titer at weeks post-vaccination^a^											
Groups	0	2	4^b^	6	8	12	16	20	24	28	32^c^	34
mE2-15 VG	<10	160	1280	10240	5120	1280	1280	1280	640	320	80	20480
mE2-206 VG	<10	320	640	20480	10240	10240	10240	10240	2560	1280	640	20480
mE2-61 VG	<10	320	640	81920	81920	81920	40960	40960	40960	20480	10240	20480
mE2-50 V2	<10	40	320	10240	5120	5120	5120	2560	2560	2560	2560	20480
PBS	<10	<10	<10	<10	<10	<10	<10	<10	<10	<10	<10	×^d^

a The neutralizing antibody titer is expressed as the reciprocal of the highest dilution of serum that could inhibit >50% of the replicates of 200 TCID_50_ of the CSFV Shimen strain.

b Pigs were booster immunized at four weeks.

c Pigs were challenged with 1×10^5^ TCID_50_ of virulent CSFV at 32 weeks post-immunization.

d Pigs exhibiting severe clinical symptoms were euthanized.

**Table 2:** Clinical outcomes of pigs following viral challenge.

			Viremia rates		
Groups	No. of pigs challenged	Fever frequency (≥40.5°C)	6 days pc	9 days pc	Survival rates
mE2-15 VG	3	4/42	0/3	0/3	3/3
mE2-206 VG	3	0/42	0/3	0/3	3/3
mE2-61 VG	3	0/42	0/3	0/3	3/3
mE2-50 V2	3	0/42	0/3	0/3	3/3
PBS	3	23/25	3/3	2/2	0/3

pc, post-challenge.

## Discussion

Vaccination is an effective way to prevent and control CSF. Although attenuated CSF vaccines are effective, widely used, and play an important role in the global control of CSF [25]–[28], they have shortcomings such as interference by maternal antibodies and the inability to differentiate from pigs infected with field virus. Reports have shown that E2 glycoprotein-based CSF subunit vaccines are safe, effective, and differentiating infected from vaccinated (DIVA) vaccines [6], [13], [16], [29]. The mammalian cell line expression system provides an alternative way to produce viral proteins with proper structures and functions [30], [31]. In this study, we described an alternative recombinant mE2 subunit vaccine candidate based on a new mammalian cell line, BCSFV-E2. BCSFV-E2 cells expressed E2 protein without the C-terminal transmembrane region and secreted it into the culture medium. As it lacked the transmembrane region, it could efficiently be secreted by the BCSFV-E2 cells into the cell culture supernatant [4], [32]. In culture conditions with spinner bottles, the secreted mE2 levels were >40 µg/mL. Further investigation is required to determine the mechanism by which BCSFV-E2 cells express E2 protein with high efficiency. The expressed mE2 protein was glycosylated and in homodimer form; these characteristics ensured the stability and antigenicity of the antigen [33]. There was only a slight decrease in the ELISA titer of the mE2 antigen in the cell culture supernatant stored at 4°C for over 12 months (data not shown).

The mE2 antigen expressed by BCSFV-E2 cells showed good immunogenicity in pigs. All the immunized pigs developed antibodies at two weeks post-vaccination, as determined by ELISA and NPLA. After booster immunization, the NPLA antibody titers increased greatly. In particular, the NPLA titer of group mE2-61 VG reached 81,920 after booster vaccination, and the high titer remained constant for at least 28 weeks (the NPLA titer was 10,240 in the mE2-61VG group). All the immunized pigs were protected from lethal challenge. Only two pigs in the mE2-15AVG group developed fever for a short duration, but recovered soon. The NPLA titers of these two pigs were 80 and 40. Bouma et al. [10] reported that an NPLA titer of 50 is sufficient for the protection of pigs from CSF. Lin et al. [6] found that neutralizing antibody titer of 32 conferred protection against CSFV infection. The NPLA titers of all group remained above 80 at 28 weeks after the booster vaccination. None of the other immunized pigs showed any clinical signs of CSF, and no viremia was detected in the vaccinated pigs. On the contrary, the control pigs exhibited the typical clinical signs of CSF after virus challenge, including high febrile response, lethargy, anorexia, and moribundity 7 to 9 days post-challenge.

Adjuvants may enhance the immunogenicity of antigens and prolong the duration of protection. In previous studies, water-in-oil-in-water double emulsions and water-in-oil emulsions were made using CSFV E2 protein expressed by baculovirus and yeast, respectively, and these vaccines were found to be effective [4], [6]. In this study, we prepared and evaluated three kinds of mE2 subunit vaccine emulsions prepared using four different adjuvants. mE2-15A VG (oil-in-water emulsion) induced an immune response quickly with high NPLA titers (1280 at 4 weeks after the first immunization) with a faster decay. mE2–61 VG (water-in-oil emulsion) induced the highest titer of NPLA antibodies with the slowest decay compared to the remaining three groups. The NPLA titer of the mE2–61 VG group was 10,240 at 32 weeks post-immunization. It was estimated that the protection conferred by this vaccine could last at least six months longer than that conferred by mE2–15 VG and mE2–206 VG. In general, for the same antigen, the vaccine emulsion was oilier, the induced immune response was stronger, and the duration of antibodies elicited by the vaccine was longer. Long-lasting immune protection is welcome for breeding gilts and boars, but immune protection for a period of 7–8 months is sufficient for growing and fattening pigs. On the other hand, the oilier emulsion has greater viscosity and was more difficult to inject; vaccinating with this emulsion will result in more side effects. Future studies on the mE2 vaccine should focus on finding the ideal balance between inducing adequate immune response and obtaining a vaccine that is convenient and has minimal side-effects. Moreover, the lowest dose of mE2 that can induce efficient immune protection needs to be determined. It would also be important to calculate the correlation between ELISA antibody titers and neutralizing antibody titers. Further related experiments will be required, including optimizing the dose of immunogen, performing challenges during longer resting periods and performing challenge studies after only a single vaccination.

In conclusion, the results of the present study demonstrate that the recombinant mE2 antigen produced by the BCSFV-E2 cells is an effective, easy to produce, and safe subunit CSF vaccine candidate.

## Supporting Information

Figure S1 **Characterization of Monoclonal antibody 12C4.** The MAb 12C4 was isotyped using Pierce Rapid Isotyping Kit. 12C4 was detected to be of IgG1 type with κ light chains (A). The MAb 12C4 specifically recognized CSFV antigen by IPMA (B). (C), Western blot analysis of MAb with *E.coli* expressed and cell line expressed E2 protein. M, protein molecular weight marker; 1, lysates of induced *E.coli* harboring E2 expressing plasmid; 2, lysates of uninduced *E.coli* harboring E2 expressing plasmid; 3, supernatant of BHK-21 cells; 4, supernatant of BCSFV-E2 cells.
(DOCX)
