# Supplementary material for: Generation and Efficacy Evaluation of Recombinant Classical Swine Fever Virus E2 Glycoprotein Expressed in Stable Transgenic Mammalian Cell Line
Source: PLoS One. 2014 Sep 8;9(9):e106891. doi: 10.1371/journal.pone.0106891 (PMC4157854; doi:10.1371/journal.pone.0106891)
Supplement: Figure S1 — Characterization of Monoclonal antibody 12C4. The MAb 12C4 was isotyped using Pierce Rapid Isotyping Kit. 12C4 was detected to be of IgG1 type with κ light chains (A). The MAb 12C4 specifically recognized CSFV antigen by IPMA (B). (C), Western blot analysis of MAb with E.coli expressed and cell line expressed E2 protein. M, protein molecular weight marker; 1, lysates of induced E.coli harboring E2 expressing plasmid; 2, lysates of uninduced E.coli harboring E2 expressing plasmid; 3, supernatant of BHK-21 cells; 4, supernatant of BCSFV-E2 cells. (DOCX) [file pone.0106891.s001.docx]

**Figure S1:**


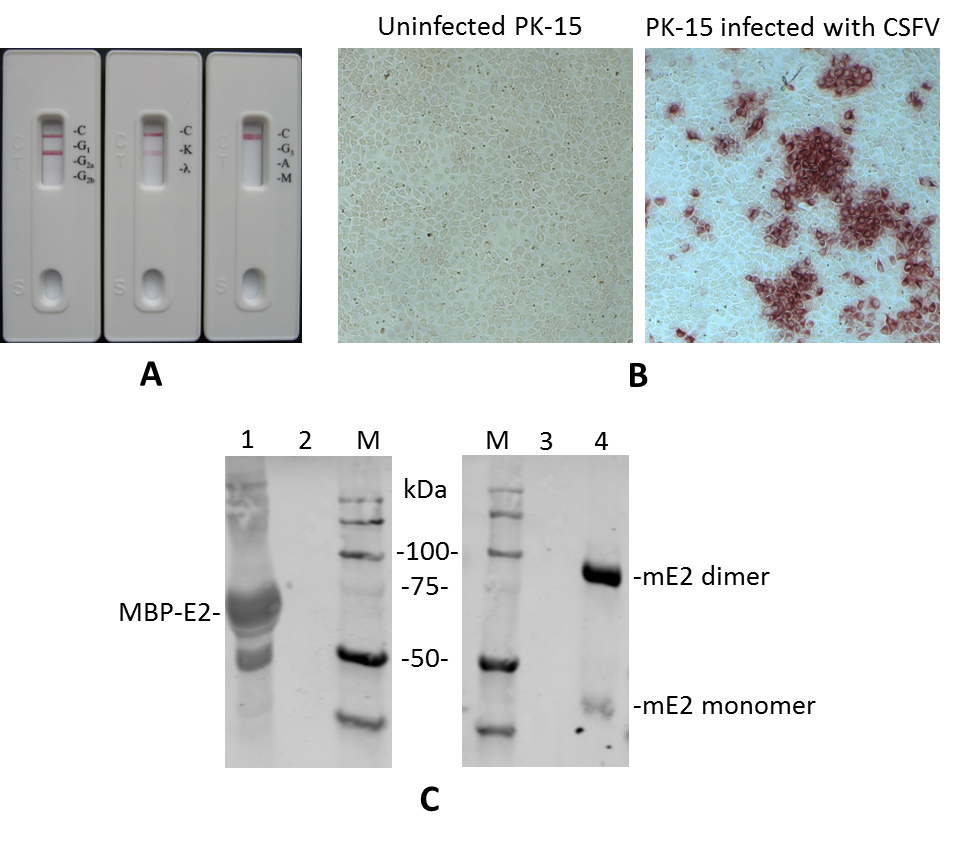


**Figure S1. Characterization of Monoclonal antibody 12C4.** The MAb 12C4 was isotyped using Pierce Rapid Isotyping Kit. 12C4 was detected to be of IgG1 type with κ light chains (A). The MAb 12C4 specifically recognized CSFV antigen by IPMA (B). (C), Western blot analysis of MAb with *E.coli* expressed and cell line expressed E2 protein. M, protein molecular weight marker; 1, lysates of induced *E.coli* harboring E2 expressing plasmid; 2, lysates of uninduced *E.coli* harboring E2 expressing plasmid; 3, supernatant of BHK-21 cells; 4, supernatant of BCSFV-E2 cells.
